# Microbial production of feedstock chemicals: An annotated selection of World Wide Web sites relevant to the topics in *microbial biotechnology*

**DOI:** 10.1111/1751-7915.13642

**Published:** 2020-08-09

**Authors:** Lawrence P. Wackett

**Affiliations:** ^1^ Department of Biochemistry, Molecular Biology & Biophysics BioTechnology Institute University of Minnesota St Paul MN 55108 USA

## Chemical feedstocks from fermentation

https://bcgc.berkeley.edu/2012/01/17/chemical-feedstock-production-by-fermentation/

This blog page contains information on the microbial biosynthesis of 1,4‐butanediol. It also has an archive list to other posts on chemical feedstocks from microbes.

## Robust microbes for chemical production

https://microbialcellfactories.biomedcentral.com/articles/10.1186/s12934-020-01325-0

This study focused on using autoclaved municipal waste as a complex feedstock for chemical production, screening different microbes for their ability to transform the mixture.

## Feedstocks – An overview

https://www.sciencedirect.com/topics/engineering/feedstocks

This page on feedstocks is a compendium and contains information related to biological and thermo‐chemical conversions of biomass.

## Synthetic biology for renewable chemicals

https://www.bio.org/sites/default/files/legacy/bioorg/docs/Synthetic-Biology-and-Everyday-Products-2012.pdf

This site from the Bio manufacturing organization discusses industrial processes for making feedstock chemicals via microbial processes.

## Fermentation‐based chemicals: Business

https://www2.deloitte.com/content/dam/Deloitte/nl/Documents/manufacturing/deloitte-nl-manufacturing-opportunities-for-the-fermentation-based-chemical-industry-2014.pdf

This report focuses on business opportunities for the fermentation‐based chemical industries.

## Biochemicals: Genomatica

https://www.genomatica.com/_uploads/pdfs/ISJ_markburke.pdf

This article deals with sustainable manufacture of chemicals with a major focus on Genomatica’s process for converting sucrose to 1,4‐propanediol.

## Maleic acid production

https://www.riken.jp/en/news_pubs/research_news/rr/20180928_2018fall_FH/index.html

Maleic acid is an important chemical, used for making surface coatings, resins, lubricants and agricultural products. This article describes a microbial process for producing the chemical.

## Electromicrobial production strategies

https://www.nature.com/articles/s41929-019-0272-0?draft=marketing

While there has been much written about using electrochemical processes to drive microbial metabolism, this article looks at the theoretical efficiencies of different processes.

## Bio‐based lactic acid

https://www.mdpi.com/2227-9717/8/2/199/htm

Lactic acid is an important chemical feedstock. One output of polylactic acid polymers, and this paper provides a techno‐economic assessment.

## Aromatic fine chemicals

https://www.imedpub.com/articles/exploring-the-microbial-production-of-aromatic-fine-chemicals-to-overcome-the-barriers-of-traditional-methods.pdf

Aromatic compounds may be produced by biological systems, via *de novo* synthesis or derived from lignin metabolism.

## Microbial production of methyl anthranilate for grape flavour

https://www.pnas.org/content/116/22/10749

Methyl anthranilate is an important industrial chemical used in foods and cosmetics. This study explored its production in genetically‐engineered *Escherichia coli* and *Corynebacterium glutamicum* strains.

## Pathways to novel chemicals

https://www.aocs.org/stay-informed/inform-magazine/featured-articles/pathways-to-novel-chemicals-february-2014?SSO=True

This site discusses the competition between bio‐based and new chemical processes for making key industrial specialty chemicals.

